# Cloud and snow detection of remote sensing images based on improved Unet3+

**DOI:** 10.1038/s41598-022-18812-6

**Published:** 2022-08-24

**Authors:** Meijie Yin, Peng Wang, Cui Ni, Weilong Hao

**Affiliations:** 1 School of Information Science and Electrical Engineering, Shan Dong Jiao Tong University, Jinan, 250357 China; 2 Institute of Automation, Shandong Academy of Sciences, Jinan, 250013 China

**Keywords:** Computer science, Information technology

## Abstract

Cloud detection is an important step in remote sensing image processing and a prerequisite for subsequent analysis and interpretation of remote sensing images. Traditional cloud detection methods are difficult to accurately detect clouds and snow with very similar features such as color and texture. In this paper, the features of cloud and snow in remote sensing images are deeply extracted, and an accurate cloud and snow detection method is proposed based on the advantages of Unet3+ network in feature fusion. Firstly, color space conversion is performed on remote sensing images, RGB images and HIS images are used as input of Unet3+ network. Resnet 50 is used to replace the Unet3+ feature extraction network to extract remote sensing image features at a deeper level, and add the Convolutional Block Attention Module in Resnet50 to improve the network’s attention to cloud and snow. Finally, the weighted cross entropy loss is constructed to solve the problem of unbalanced sample number caused by high proportion of background area in the image. The results show that the proposed method has strong adaptability and moderate computation. The mPA value, mIoU value and mPrecision value can reach 92.76%, 81.74% and 86.49%, respectively. Compared with other algorithms, the proposed method can better eliminate all kinds of interference information in remote sensing images of different landforms and accurately detect cloud and snow in images.

## Introduction

With the continuous development of remote sensing technology, satellite has become an important part of a country's economic development. According to statistics, there are more than 2,500 satellites in orbit around the world. With the return of more and more remote sensing images, these images in the investigation of land water resources, land resources, vegetation resources, environmental monitoring and other fields are playing a more and more obvious role. However, about 60% of the earth is covered by clouds on average^1^, clouds not only block ground objects, but also affect the accuracy and reliability of inversion of surface and atmospheric parameters, which is an unfavorable factor restricting the application of remote sensing images. Therefore, it is necessary to accurately detect the clouds in remote sensing images to facilitate the subsequent analysis and interpretation of remote sensing images. However, cloud and snow have similar color and texture features in remote sensing images, it is necessary to distinguish them accurately in the detection process to improve the accuracy of cloud detection.

Classical cloud detection methods mainly include physical threshold method, method based on cloud texture and spatial characteristics and method based on machine learning. With the development and application of deep learning, deep learning methods based on CNN, FCN and Unet are gradually applied to remote sensing image cloud detection. The imaging sensor technology carried by early satellites is not mature, and remote sensing images had few spectral segments, so physical threshold-based methods such as OTSU^2,3^ and CLAVR^4^ are mostly used for cloud detection. With the development of imaging sensors, the bands and spectral segments of remote sensing images are increasing, people begin to use the physical characteristics of clouds, such as highlighting, high reflectivity and low temperature, to complete cloud detection by filtering physical radiation threshold^5–7^. The above thresholding method can detect large and thick clouds in remote sensing images, but it is easy to be disturbed by white ground objects such as ice and snow. The cloud detection method based on cloud texture^8,9^ expresses the difference between cloud and surface objects in texture features through gray level co-occurrence matrix and improved fractal dimension, so as to realize remote sensing image cloud detection. The cloud detection method based on image spatial information^10^ takes advantage of the fact that the radiation changes degree of cloud covered area is higher than that of other areas, and compares the change of regional radiation value of each pixel in the image with the set threshold value to achieve pixel-level cloud detection. The method based on cloud texture and spatial characteristics is mainly applicable to remote sensing images with a single background such as ocean and plain, with a small scope of application and low detection accuracy for remote sensing images with complex background. With the development of machine learning, methods such as decision tree and support vector machine (SVM)^11,12^ have been applied to remote sensing image cloud detection. Fmask algorithm^13,14^ separates potential cloud pixels and clear sky pixels based on cloud physical properties, masks cloud generation probability on land and water through normalized temperature probability, spectral variability probability and brightness probability, and then deduces potential clouds. ACAA algorithm^15^ reduces the influence of cloud variability on cloud amount by scanning remote sensing image twice, and then performs cloud detection through decision tree threshold. Reference^16^ uses vector machines to create a decision boundary for cloud detection through the representation of mapping data. In addition, the elevation-assisted cloud and snow detection method^17,18^ compares the elevation differences between cloud, snow and other ground objects, and uses multiple image intensive matching technology to obtain three-dimensional geometric features of cloud, so as to realize the distinction between cloud and ground. The above cloud detection method based on machine learning is highly dependent on training data and uses artificially designed features. The rationality of feature design will have a great influence on detection results, and the method has a small scope of application.

With the improvement of computer performance, deep learning algorithm develops rapidly in image segmentation, object detection and other fields. As the basis of deep neural network, CNN network has been widely used in image classification and target detection. Its advantage lies in that it can learn deeper and more abstract image features by increasing the number of convolutional layers and increasing the receptive field. Reference^19^ uses a simple CNN network to complete the cloud detection task, which solves the problem of inaccurate detection results of threshold method due to few bands and limited spectral range of remote sensing images. However, the network structure used by this method is simple and the generalization ability is poor. Reference^20^ proposed a method combining clustering and convolutional network to realize cloud detection. Firstly, super pixels are obtained by SLIC clustering, and then feature information of cloud is extracted by convolutional neural network, so as to accurately extract cloud boundary, but end-to-end cloud detection cannot be achieved. Reference^21^ proposed a robust multi-scale segmentation method based on deep learning, which utilized spectral spatial features of the remaining convolution layer to carry out feature mapping, and then proposed a new loss function for cloud and cloud shadow target extraction. Reference^22^ proposed a cloud detection method based on multi-scale characteristic convolutional neural network (MF-CNN). By stacking multi-band spectral information, high-level semantic information is combined with low-level spatial information to accurately distinguish thick cloud, thin cloud and non-cloud regions. Reference^23^ proposed a dual-branch PCA network (PCANet) to detect cloud pixels by extracting high-level semantic information of remote sensing images and combining it with SVM classifier. Reference^24^ introduced high-frequency feature extractors and multi-scale convolution in Unet networks to refine cloud boundaries and predict debris clouds. Reference^25^ proposed a cloud and snow detection method combining Resnet50 and Deeplabv3+ with full convolutional neural network, experimental results show that this method does not have a high distinction between cloud and snow and is prone to misjudgment. Reference^26^ proposed a cloud detection method based on spectral library and convolutional neural network, which can accurately detect thin cloud and broken cloud by using residual learning and one-dimensional CNN network to accurately capture spectral information, however, this method ignored spatial information and is limited in feature extraction of complex scenes with mixed spectral information of ground and thin cloud. Reference^27^ proposed a cloud detection method for high-resolution remote sensing images based on convolutional neural network, which uses unsupervised learning method for pre-training to obtain cloud feature information in remote sensing images to achieve cloud detection, however, this method ignores cloud texture and other feature information, resulting in low detection accuracy. The cloud detection method based on deep learning can automatically extract cloud feature information without designing features beforehand, and cloud detection has high accuracy. However, different types of remote sensing images have great differences in color information and spatial resolution, and remote sensing images have complex landforms, including snow, ocean, plain, mountain and other landforms, which requires cloud detection methods based on deep learning to have strong generalization ability.

This paper adopts the deep learning method and takes Unet3+ as the backbone network to accurately detect clouds and snow in remote sensing images. The main contributions are as follows: (1) Carry out color space conversion for remote sensing images, and use RGB and HIS remote sensing images as inputs of Unet3+ network to improve the network's discrimination of cloud and snow features; (2) Resnet50 is used as the feature extraction network of Unet3+ to deepen the network structure and extract deeper feature information of remote sensing images, while avoiding the problems of gradient explosion and gradient dispersion, and convolutional attention mechanism is introduced in Resnet50 network to strengthen the network's attention to cloud and snow area, and reduce the unnecessary calculation overhead caused by high proportion of background area in cloud and snow detection process; (3) Weighted cross entropy loss is designed to avoid the problem that the model weight gradually deviates to the background due to the excessive background class pixels in remote sensing images during network model training, so as to further improve the efficiency and accuracy of cloud and snow detection.

The rest work of this paper is as follows: “Correlation network model” section introduces the structure of the network model used, including Unet3+ network and Resnet50 network; “Methodology” section describes many details of the proposed method, including the improvement of network structure, the optimization of network input and the design of loss function. “Experimental results and analysis” section is the comparison and analysis with other cloud detection methods under the same data set. The last section summarizes the work of this paper and puts forward the next research direction.

## Correlation network model

### Unet3+ network

**Figure 1:**
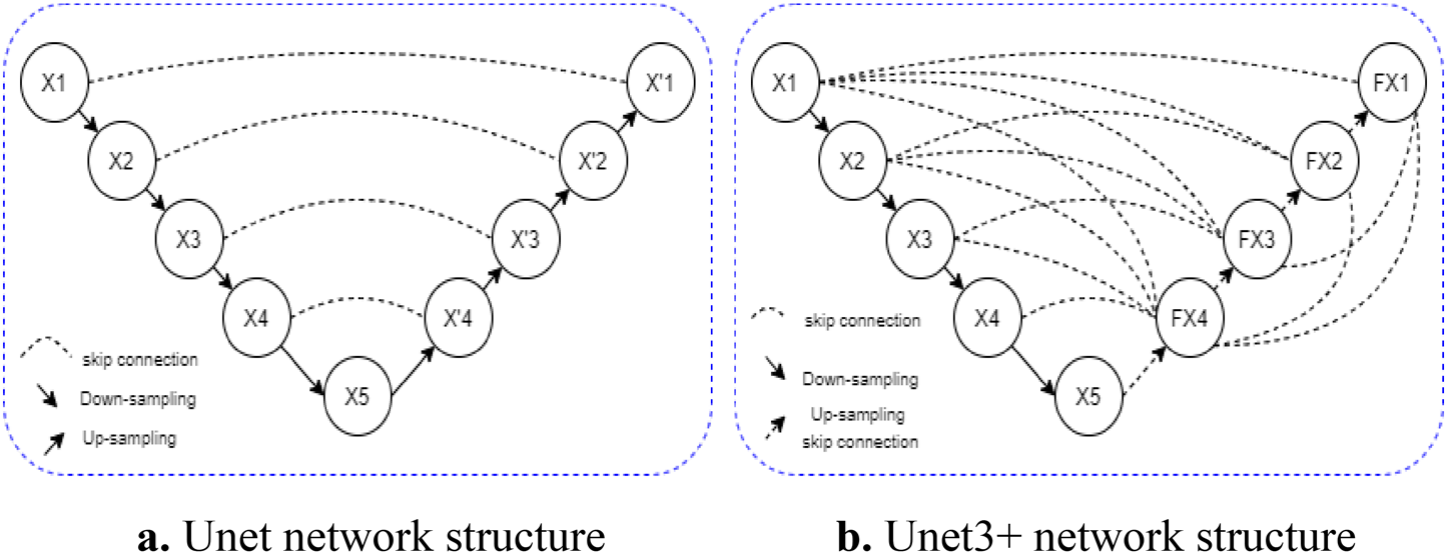
Schematic diagram of Unet and Unet3+ network structure.

Unet network is a deep learning model based on encoder-decoder structure, it is originally used in medical image processing and has been widely used in remote sensing image segmentation in recent years. Unet network only fuses feature maps of the same scale in the decoder part, and lacks sufficient information to explore from the full scale, so the location and boundary of learning objectives cannot be defined. In order to remedy the above defects, Unet3+ network^28^ came into being. Unet network connects encoders and decoders of the same scale, while Unet3+ network fuses feature maps of different scales through nested and dense jump connections. As shown in Fig. 1. Figure 1a is the network structure of Unet, and Fig. 1b is the network structure of Unet3+ . As shown in Fig. 1b, X1, X2, X3, X4 and X5 are feature maps generated after feature extraction network of each layer, and then feature maps of the same scale as X4 are obtained through different pooling operations. Then, through the convolution operation of 3 × 3 filters with the same number of channels as X4, the feature graph with the same number of channels as X4 is obtained, and then the fusion feature graph FX4 is obtained by splicing and fusion with X4. By analogy, fusion feature graphs FX3, FX2 and FX1 are obtained through the same operation.

In Unet3+ network, feature maps of different scales describe different image feature information. The shallower the network, such as FM1, the smaller the receptive field, the stronger the geometric detail representation ability of the extracted shallow feature information, and the more able it is to capture the spatial information of the object, such as the contour and edge of the object. However, the deep network, such as FM4, has a large receptive field and strong semantic representation ability of extracted deep feature information, which can determine object attributes, but cannot highlight details such as spatial geometric features of objects. When remote sensing images enter the network, after a series of pooling operations, the spatial details of cloud and snow in the image will be gradually lost, but the semantic representation ability of cloud and snow is constantly enhanced. In this paper, Unet3+ network is adopted to fully extract multi-scale feature information of cloud, snow and other ground objects in remote sensing images, and full-scale feature fusion method is adopted to fuse shallow feature information and deep feature information, so as to avoid feature information dilution in pooling convolutional operation and improve the detection accuracy of cloud and snow.

### Resnet50 network

**Figure 2:**
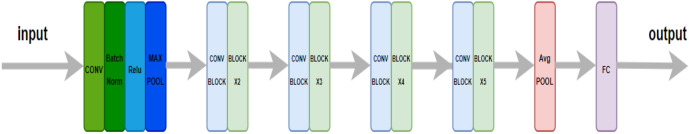
Schematic diagram of Resnet50 network structure.

Resnet50 is a deep residual network^29^, which contains 49 convolutional layers and 1 full connection layer, as shown in Fig. 2. The Resnet50 network architecture can be divided into seven parts, the first part is about convolution, regularization, activation, and maximum pooling of the input. The second, third, fourth and fifth parts all contain residual modules, which transfer the network input across layers and carry out equal mapping, and then add the results of the convolution operation. The sixth part is global average pooling, transforming the convolution calculation results of the first five parts into a feature vector. The seventh part is the full connection layer, which uses the classifier to calculate the feature vector and output the category probability.

In this paper, Resnet50 network is taken as the feature extraction network of Unet3+ , by increasing the number of convolutional network layers, the cloud and snow in remote sensing images in color, texture and other deeper feature information can be extracted. Due to the application of residual module in Resnet50 network, the number of parameters is greatly reduced, and problems such as gradient explosion and gradient dispersion caused by too many network layers can be effectively avoided, thus improving the robustness of cloud detection.

## Methodology

**Figure 3:**
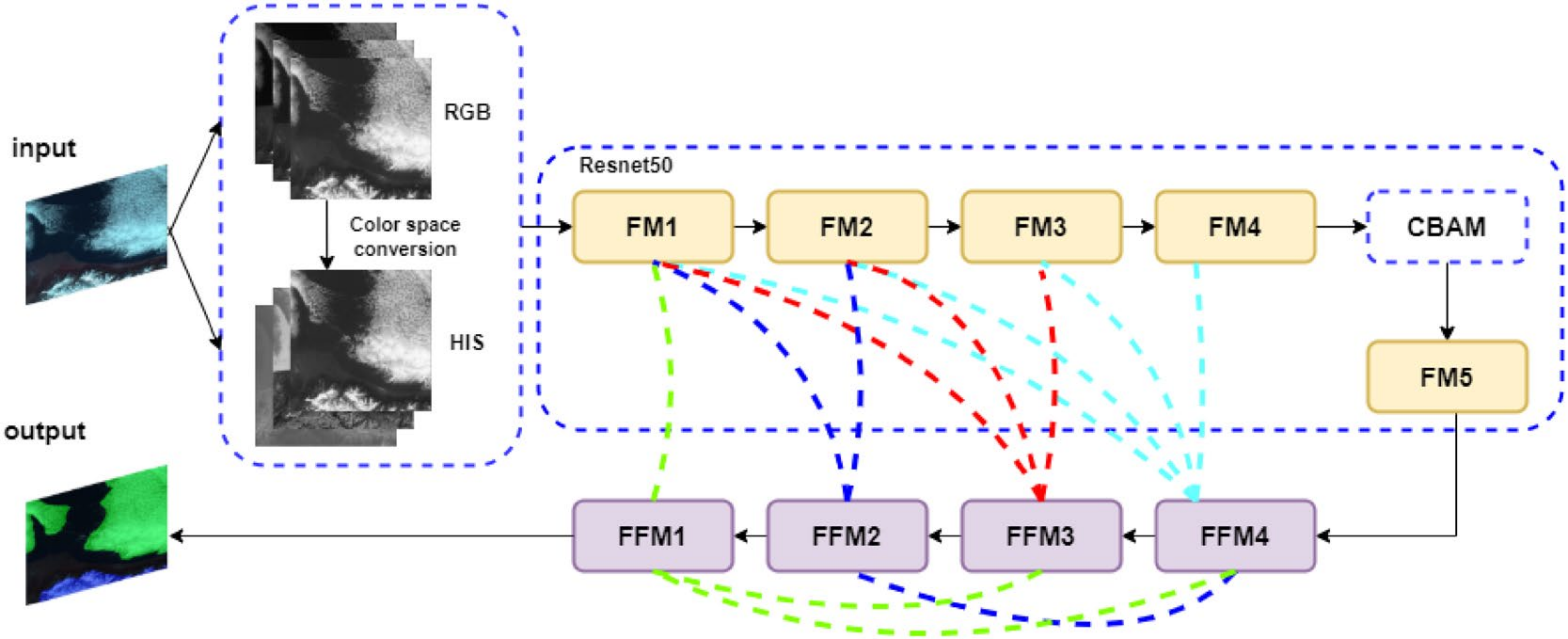
Flowchart of the method presented in this paper.

Based on Unet3+ network structure, this paper proposes an accurate cloud and snow detection method, and the process is shown in Fig. 3. Firstly, the remote sensing image is converted into color space as the input of the network. Resnet50 is taken as the feature extraction network of Unet3+ and added into CBAM to strengthen the network’s attention to cloud and snow. The weighted cross entropy is constructed to increase the weight of cloud and snow in remote sensing images, so as to avoid the model bias in the training process.

### Multidimensional image input

**Figure 4:**
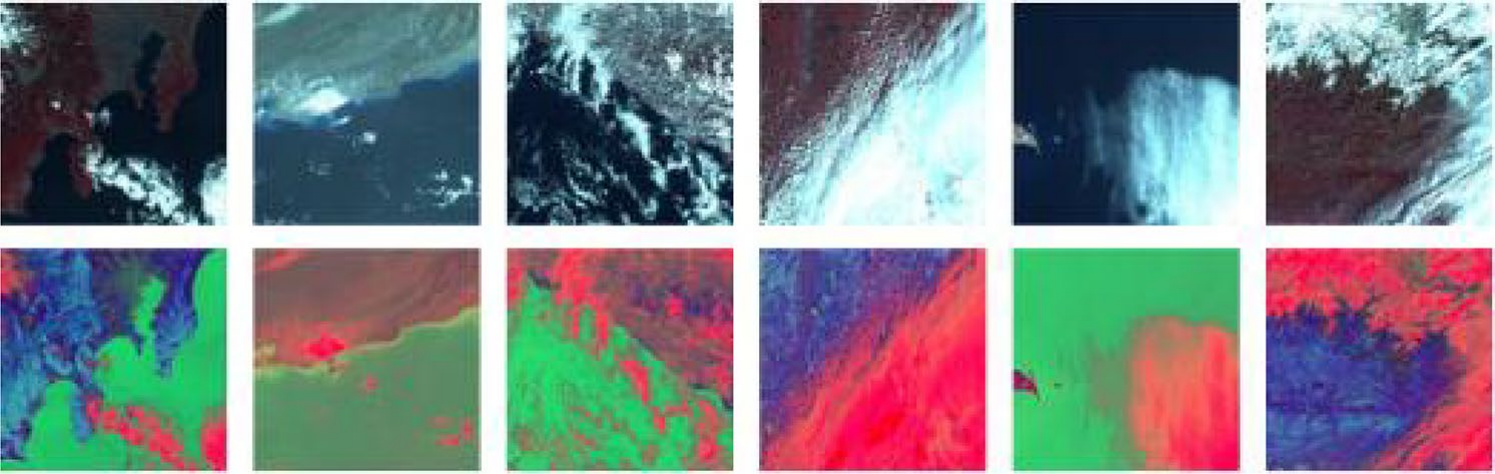
Schematic diagram of color space conversion results. Among them, the first behavior RGB image, the second behavior transformed HIS image.

Most semantic segmentation networks use RGB images as input to extract feature information of R, G and B channels, however, RGB color space is sometimes not a good clustering basis for image clustering, especially for remote sensing images rich in color information. In remote sensing images, cloud and snow have great similarity in color and texture, and it is difficult to distinguish cloud and snow in ordinary RGB images from the naked eye. Feeding the images into the network makes it harder to distinguish between cloud and snow features. HIS color space based on human visual system, with Hue, Intensity and Saturation to describe the image, the color information and gray information of the image are separated in order to express the color category and the degree of the same color in a deeper level. In the case of highly similar objects, such as white clouds and snow, it is possible to distinguish the types of objects in more detail. In this paper, based on RGB images, color space conversion is carried out to convert them into HIS images, and the conversion formula is shown in (1)–(3). The color space conversion results of some images are shown in Fig. 4.

1 $$\left\{ {\begin{array}{*{20}l} {H = \left\{ {\begin{array}{*{20}l} {\theta ,} \hfill & {G \ge B} \hfill \\ {2\pi - \theta ,} \hfill & {G < B} \hfill \\ \end{array} } \right.,} \hfill \\ {where\;\theta { = }\cos^{ - 1} \left( {\frac{(R - G) + (R - B)}{{2\sqrt {(R - G)^{2} + (R - B)(G - B)} }}} \right)} \hfill \\ \end{array} } \right.$$

2 $$S = 1 - \frac{3\min (R,G,B)}{{R + G + B}}$$

3 $$I = \frac{R + G + B}{3}$$

Finally, RGB images and HIS images are used as the input of Unet3+ network to extract the features of cloud, snow and other ground objects respectively, so as to improve the network’s discrimination of cloud and snow features.

### Feature extraction network and convolutional attention module

**Figure 5:**
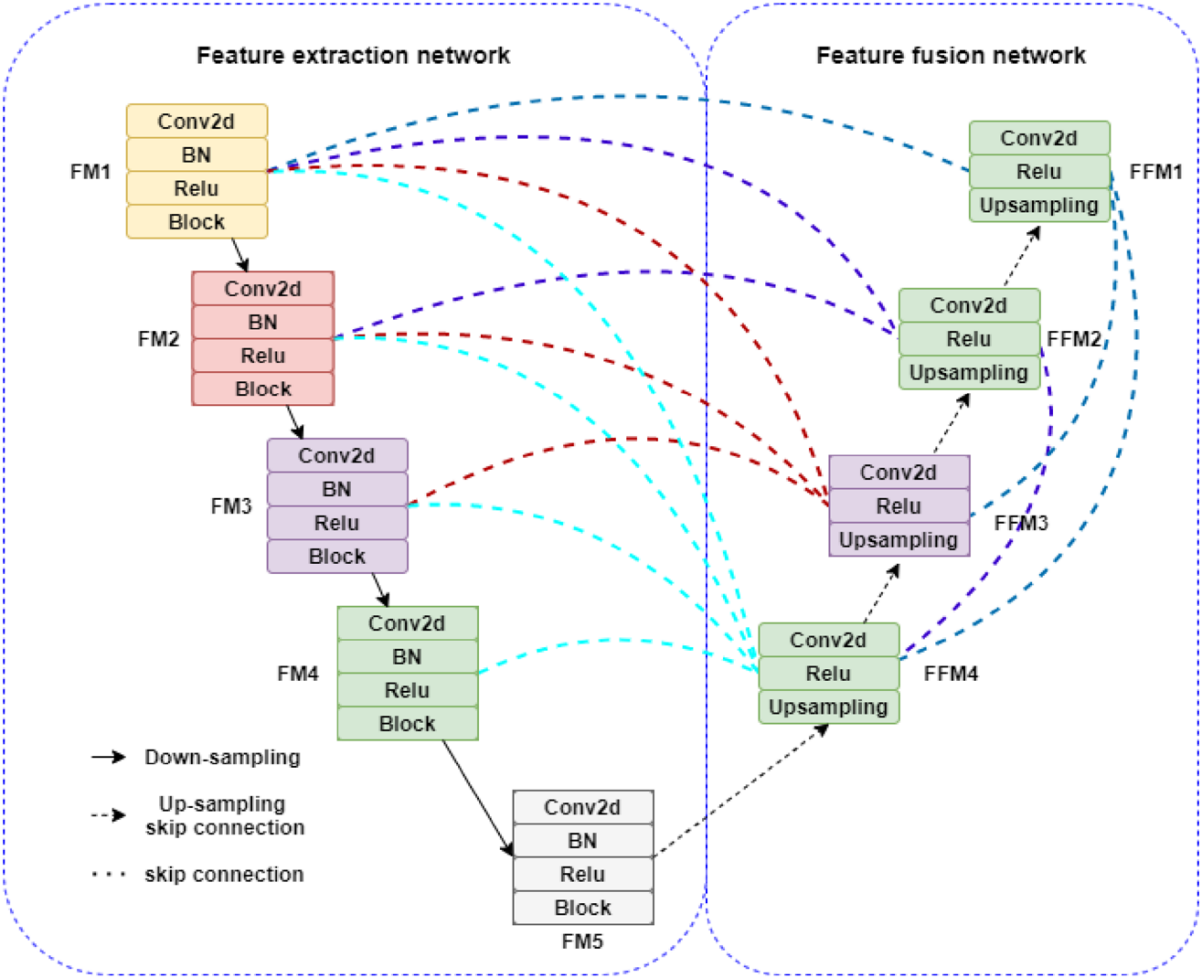
Unet3+ network structure after the introduction of Resnet50.

In this paper, Resnet50 network is used to replace the feature extraction network of Unet3+, as shown in Fig. 5. The feature extraction network of Unet3+ contains five encoders, each of which contains three effective convolutional layers and a maximum pooling layer. The improved feature extraction network contains five layers of encoders, each of which contains several convolutional layers with different convolutional kernels, regularization layers, ReLU activation layers and residual modules, five effective feature graphs FM1-FM5 are obtained through different convolutional operations. Each decoder layer contains convolution layer and activation function layer, and the effective feature images of different scales are fused by jump connection respectively to obtain fusion feature images FFM1-FFM4.

**Figure 6:**
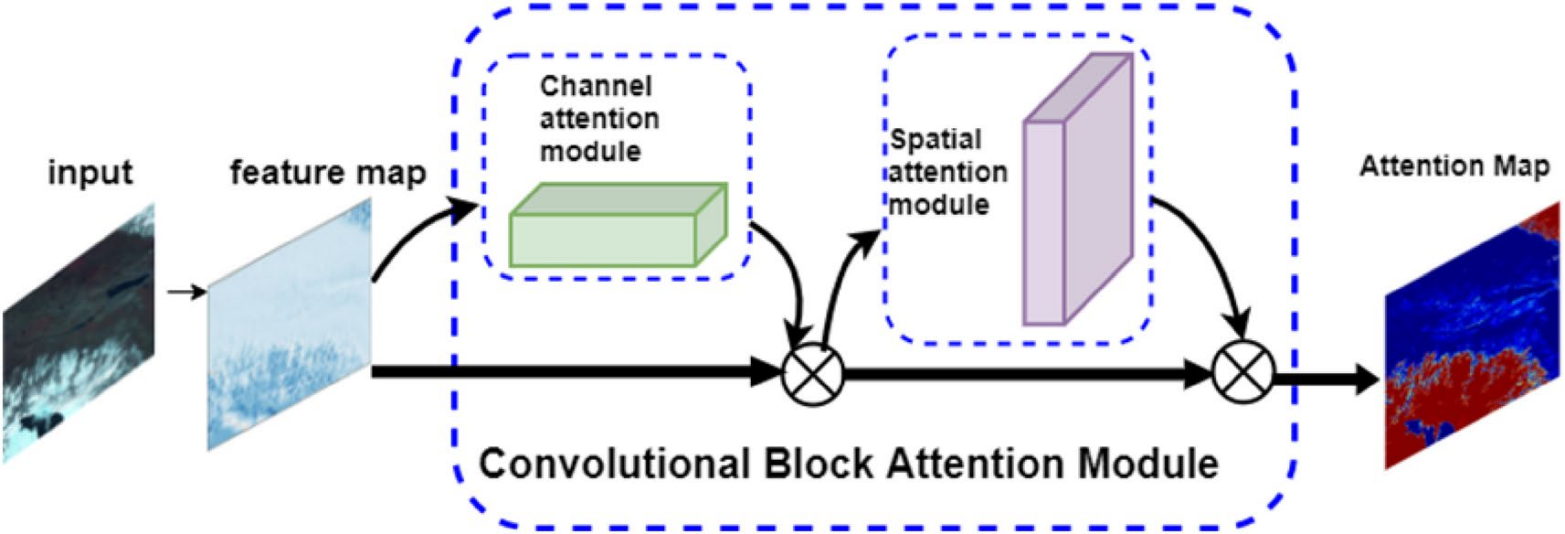
Schematic diagram of CBAM.

Due to the wide coverage and rich color information of remote sensing images, the network will inevitably invest a lot of computing power in non-cloud and non-snow areas during feature extraction, which reduces the network work efficiency. In order to increase the network's attention to cloud and snow and extract the characteristic information of cloud and snow at a deeper level, convolutional block attention module (CBAM)^30^ is added to Resnet50 network, as shown in Fig. 6, which mainly includes channel attention mechanism and spatial attention mechanism. The mechanism of channel attention is that the input feature maps are respectively processed by global maximum pooling and global average pooling based on width and length, and then processed by multi-layer perceptron (MLP). The features of MLP output are added up, and finally activated by sigmoid to generate channel attention feature maps. The channel attention feature map is multiplied with the input image as the input feature map of the spatial attention module, and the expression is shown in (4). The spatial attention mechanism is to make a global maximum pooling and global average pooling based on channel and merge the results based on channel. Then, a convolution operation is performed to reduce the dimension to one channel, and then activated by sigmoid to generate the spatial attention feature graph, as shown in (5). Finally, the final feature map is obtained by multiplying the channel attention feature map and the spatial attention feature map. By adding CBAM, the network can enhance the attention of cloud and snow in remote sensing image, and extract the features of cloud and snow with less computational overhead

4 $$\begin{aligned} M_{c} \left( {\text{F}} \right) & = \sigma ({\text{MLP}}({\text{AvgPool}}\,\left( {\text{F}} \right)) + {\text{MLP}}({\text{MaxPool}}\,\left( {\text{F}} \right))) \\ & = \sigma ({\text{W}}_{1} ({\text{W}}_{0} ({\text{F}}_{avg}^{{\text{c}}} )) + {\text{W}}_{1} ({\text{W}}_{0} ({\text{F}}_{\max }^{{\text{c}}} ))) \\ \end{aligned}$$

5 $$\begin{aligned} M_{s} \left( {\text{F}} \right) & = \sigma \left( {{\text{f}}^{7 \times 7} \left( {\left[ {{\text{AvgPool}}\,({\text{F}});\;{\text{MaxPool}}\,({\text{F}})} \right]} \right)} \right) \\ & = \sigma \left( {{\text{f}}^{7 \times 7} \left( {\left[ {{\text{F}}_{avg}^{s} ;\;{\text{F}}_{\max }^{s} } \right]} \right)} \right) \\ \end{aligned}$$

where MLP represents multi-layer perceptron, F represents feature graph, $\sigma$ represents sigmoid activation function, W_1_ and W_0_ represent the shared weight of two inputs of multi-layer perceptron respectively, f^7×7^ represents the size of convolution kernel of 7 × 7.

**Figure 7:**
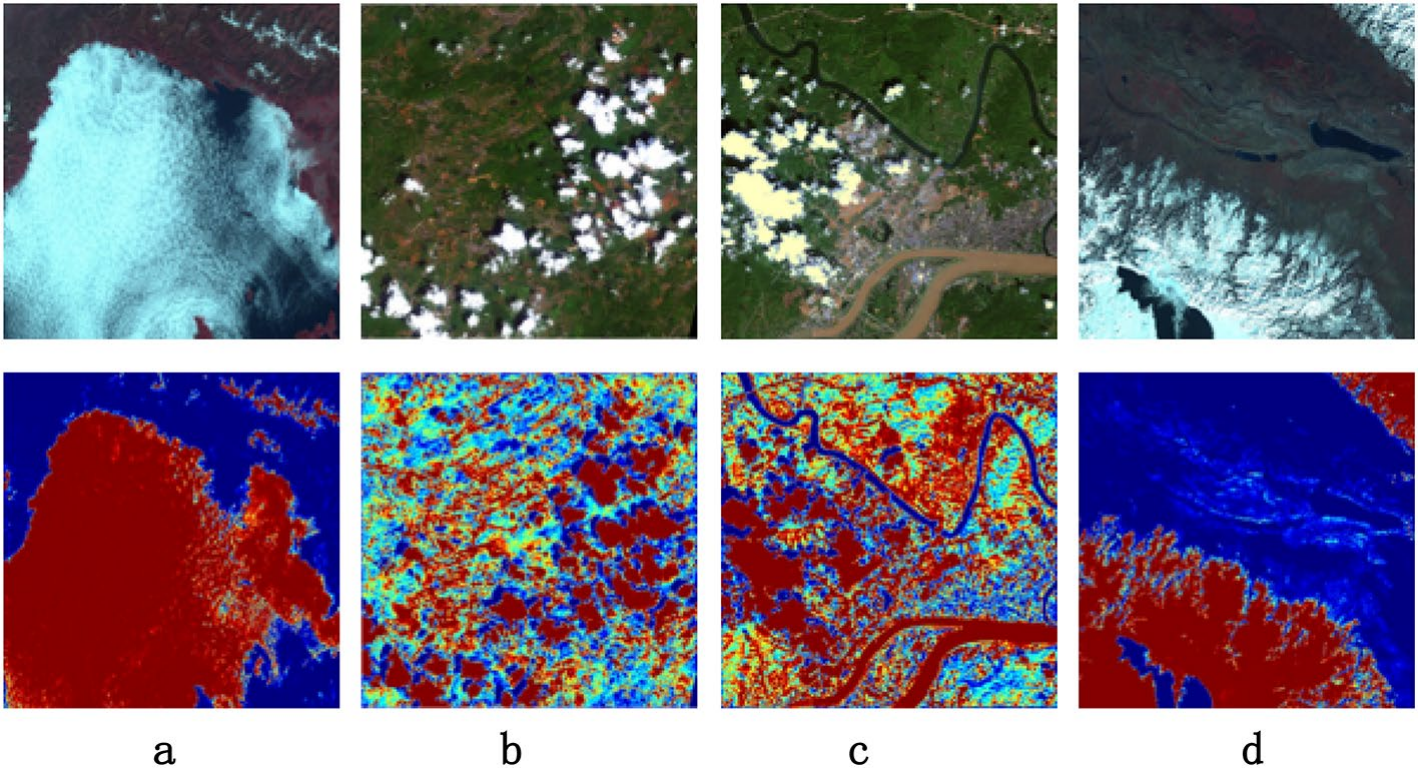
Schematic diagram of CBAM output results. Wherein, the original remote sensing image of the previous act and the CBAM output result corresponding to the next act. The areas in dark red indicate the areas where the network is concerned.

In order to verify the effectiveness of CBAM, this paper outputs the attention map of remote sensing images including frozen soil, mountains, towns and snowfields, the results are shown in Fig. 7, and the dark red area represents the key area concerned by the network. In order to verify the effectiveness of CBAM, this paper outputs the attention map of remote sensing images including frozen soil, mountains, towns and snowfields. The results are shown in Fig. 7, and the dark red area represents the key area concerned by the network. As can be seen from the figure, after network training, for the image with relatively single texture shown in Fig. 7a and Fig. 7d, the attention of the network is mainly focused on the cloud and snow areas in the figure. For images with more complex textures as shown in Fig. 7b,c, the attention of the network covers a wider area, but the cloud and snow areas are all within the attention range of the network.

### Weighted cross entropy loss

Loss function is an important link in neural network, which is used to express the difference between the predicted result and the actual result. In classification networks and semantic segmentation networks, cross entropy is often used as the loss function^31^, as shown in Formula (6):

6 $$L = - \sum\limits_{{{\text{c}} = 1}}^{M} {y_{c} } \log (p_{{\text{c}}} )$$

where c represents the predicted category, M represents the number of categories; y_c_ represents the one-hot vector, if c is consistent with the actual type, the value is 1, otherwise, the value is 0; *P*_c_ represents the probability that the predicted sample is in the c category.

At present, cross entropy loss function has been widely used in semantic segmentation network models. When the cloud and snow is far less than the number of pixels in remote sensing image background pixel number, if you use the cross entropy as the loss function, in the process of network model training, the number of y_c_ = 0 in the formula (6) will be far greater than the number of y_c_ = 1, which results in the loss function of y_c_ = 0 composition is dominant, weights of network model will be seriously biased towards the background pixels, It affects the accuracy of cloud and snow detection.

In order to avoid the problem that the weight of the network model gradually deviates to the background due to too many background pixels in the process of cloud and snow detection, this paper designs a new weighted cross entropy loss based on the original cross entropy loss function, as shown in Formula (7):

7 $$L = - \sum\limits_{{{\text{c}} = 1}}^{M} {{\text{w}}_{{\text{c}}} y_{c} } \log (p_{{\text{c}}} )$$

where w_c_ represents the weight coefficient of category C, and the calculation method is shown in Formula (8):

8 $${\text{w}}_{{\text{c}}} = {\text{exp}}\left( { - \frac{{N_{C} }}{N}} \right)$$

where $N_{{\text{c}}}$ represents the number of pixels of category C, *N* represents the total number of pixels of a remote sensing image.

w_c_ represents the weight coefficient of category c, the size of the weight coefficient is related to the number of pixels of category c, and the more pixels of category c, the smaller the weight coefficient is to ensure the balance of categories. However, the weight coefficient should not be too large or too small to prevent problems such as category imbalance. Therefore, exp function is adopted in this paper to linearly normalize the weight coefficient and limit the weight coefficient within the range of (e^−1^, 1), by controlling the size of the weight coefficient w_c_, prevent network model bias caused by too large or too small weight of a certain type of pixel. In addition, exp function increases monotonically in the interval of (e^−1^, 1). When the number of class c pixels increases, $- \frac{{N_{{\text{c}}} }}{N}$ decreases and the weight coefficient decreases, which can effectively weaken the influence of high frequency classes in data samples and solve the problem of unbalanced data samples. In the process of cloud and snow detection of remote sensing images, the more pixels of the background category, the smaller the weight coefficient will be, making the model weight biased towards the cloud and snow region, enabling the network to learn the characteristic information of cloud and snow better, improving the robustness of the model and improving the accuracy of cloud and snow detection.

## Experimental results and analysis

### Construction of cloud and snow detection data-set

**Figure 8:**
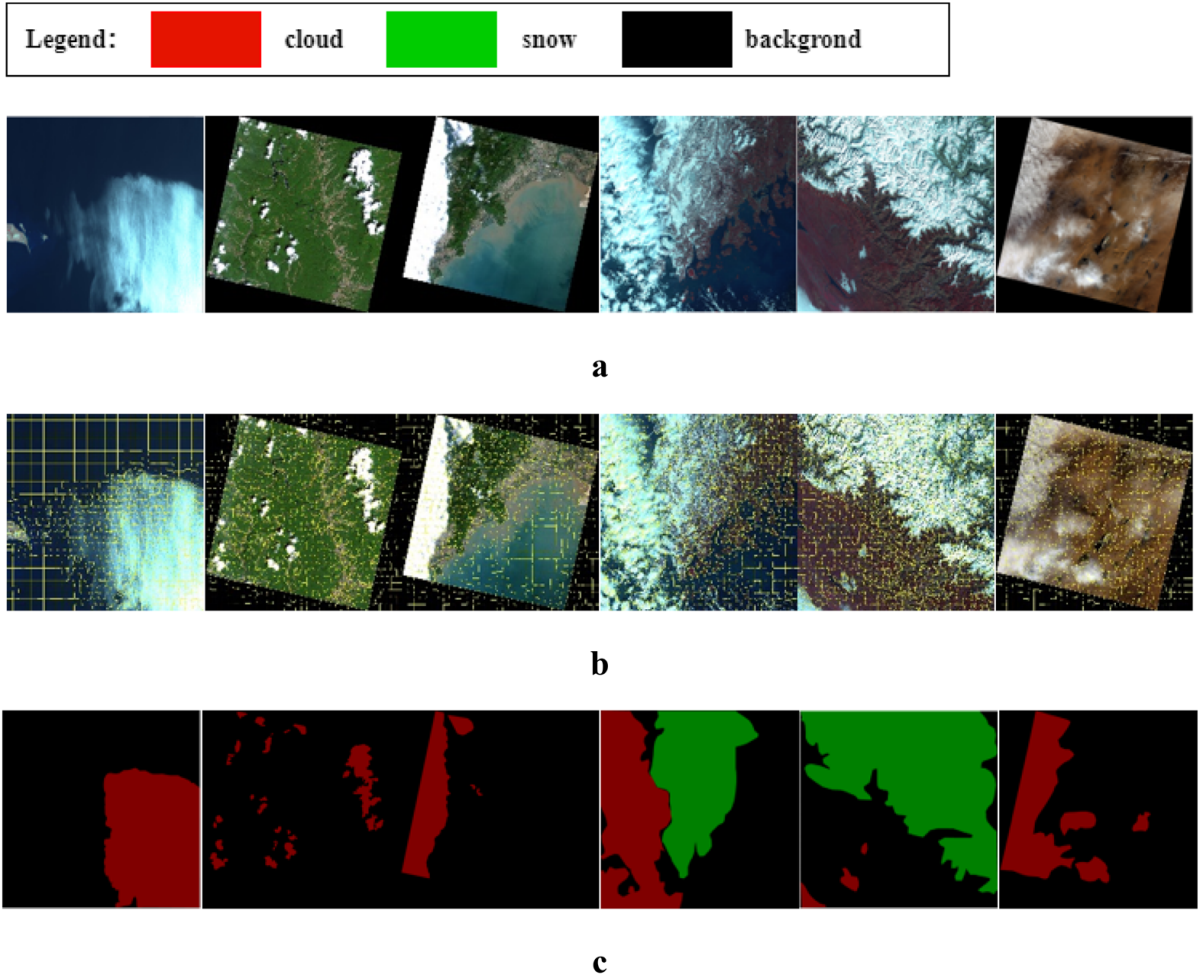
Labels of remote sensing images of different landforms obtained after superpixel segmentation. (**a**) is the original remote sensing image, (**b**) is the result of super pixel segmentation, (**c**) is the label image.

In this paper, remote sensing images taken by Gaofen-2 (GF-2) and Huanjing-1 (HJ1A) satellites are used to construct a dataset. GF-2 imagers the ground landscape by pushing and scanning, and can take panchromatic and multispectral images with high resolution, high positioning accuracy and high radiation quality. HJ1A satellite is mainly used for environment and disaster monitoring and prediction, with CCD camera and Hyperspectral imager. In order to test the effectiveness and accuracy of cloud and snow detection method proposed in this paper, remote sensing images of various landforms are selected, as shown in Fig. 8a, including ocean, plain, town, frozen soil, snow and desert, etc. When making the cloud and snow detection data set, due to the diverse shapes and irregular spatial distribution of cloud and snow in the image, in order to improve the annotation accuracy, this paper first adopts the simple linear iterative clustering method (SLIC)^32,33^ to perform super-pixel segmentation on remote sensing image, as shown in Fig. 8b, and determines the contour of cloud and snow at the sub-pixel level. Then, by manual labeling, cloud labels and snow labels are respectively labeled according to the outlined cloud and snow contours, as shown in Fig. 8c, which effectively reduces labeling errors and makes the produced data set more accurate. The label diagram contains three types of labels: red for cloud, green for snow, and black for background. Finally, the completed cloud and snow detection data set is processed with data enhancement, and a total of 5000 images are obtained. Among them, 4500 images are used as training set and 500 images are used as verification set, and the ratio of training set to verification set is 9:1.

### Comparison of feature extraction network experiments

Resnet50 can extract deep feature information of image with high precision and moderate computation. In this paper, Resnet50 is used as the feature extraction network of Unet3+, which can accurately extract the feature information of cloud and snow from remote sensing images. In the constructed cloud and snow detection data set, the experimental comparison of VGG16, Resnet34, Resnet50 and Resnet101 is carried out respectively. Mean intersection over union (mIoU), mean pixel accuracy (mPA), mean precision (mPrecision) and Estimated Total Size are used as evaluation indexes, and the results are shown in Table 1. Among them, IoU, PA and Precision are common evaluation indexes in semantic segmentation, which are used to measure the similarity between segmentation results and real images. Their formulas are shown in (9), (10) and (11).

9 $$I{\text{oU}} = \frac{TP}{{TP + FN + FP}}$$

10 $$PA = \frac{TP + TN}{{TP + FP + FN + TN}}$$

11 $$Precision = \frac{TP}{{TP + FP}}$$

**Table 1:** Comparison of experimental results of feature extraction network.

Backbone network	mPA (%)	mIoU (%)	mPrecision (%)	Estimated total size (M)
VGG16	86.97	72.94	78.43	2907.95
Resnet34	87.45	71.47	77.37	1250.33
Resnet101	89.45	77.63	84.32	3437.68
Resnet50	89.70	78.72	84.59	2741.59

In the formula, TP represents the number of correctly classified positive class pixels, FP represents the number of correctly classified negative class pixels, FN represents the number of incorrectly classified positive class pixels, TN represents the number of accurately classified negative class pixels.

As shown in the table, compared with other backbone networks, Resnet50 has the highest evaluation index and a moderate number of Estimated Total Size.

**Figure 9:**
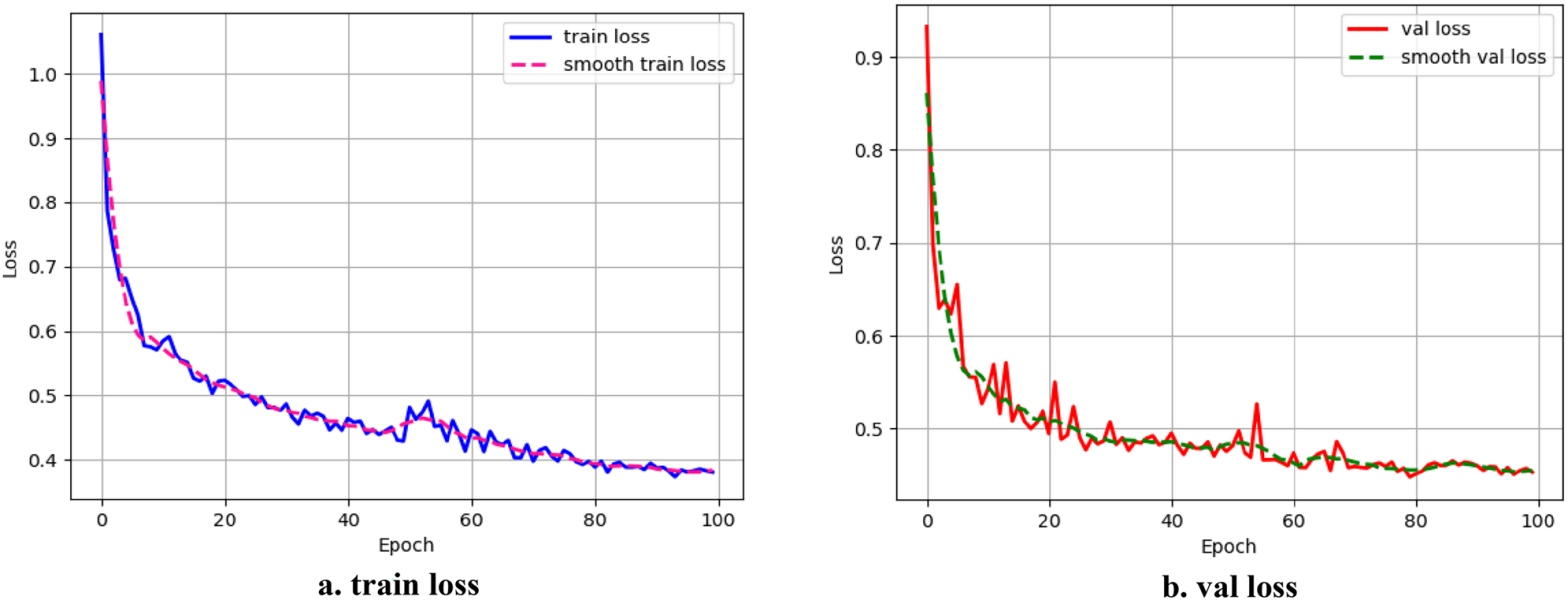
Schematic diagram of network model training process used in this paper.

In this paper, the deep learning framework of PyTorch^34^ is used to train and test the network model. The compilation environment is conda 4.12.0, the CPU is i7-10,700, 16 GB RAM, NVIDIA GeForce RTX 3070-8G and CUDA 11.2. In the experiment, batch-size is set to 8, initial learning rate is set to 0.01, and Adam optimizer is used to optimize the network. Adam optimizer is a first-order optimization algorithm that can replace the traditional stochastic gradient descent process. It can update the weight of neural network iteratively based on training data and adjust the gradient descent adaptively according to the size of learning rate. It has the advantages of simple implementation, high computational efficiency, less memory requirement, and the updating of parameters is not affected by the scaling transformation of gradient. Moreover, the hyperparameters have good interpretation, and usually need no adjustment or only fine tuning. To ensure good learning efficiency, every 50 epochs, the learning rate is divided by 10 to train a total of 100 epochs. The training process is shown in Fig. 9. It can be seen from the figure. that when the training arrived at the 70th epoch, the network model had tended to fit.

### Ablation experiment

In order to test the improvement of cloud and snow detection performance by each method described in “Methodology” section, ablation experiment is conducted in this paper, and the results are shown in Table 2.

**Table 2:** Comparison of ablation results.

Method	mPA (%)	mIoU (%)	mPrecision (%)	Estimated total size (M)
Resnet50	89.70	78.72	84.59	2741.59
Resnet50 + multidimensional image input	90.24	78.89	85.27	2741.59
Resnet50 + multidimensional image input + full-scale feature fusion and CBAM	91.62	79.73	85.68	2787.46
Resnet50 + multidimensional image input + full-scale feature fusion and CBAM + weighted cross entropy loss	92.76	81.74	86.49	2787.46

Multidimensional image input: in the feature extraction stage, the input of the original network is RGB image, and the feature extraction of cloud and snow is insufficient. After HIS image is added, deeper color, texture and other feature information can be extracted, which improves the final cloud and snow detection result. The experimental results are shown in Table 2. mPA value, mIoU value and mPrecision value are improved by 0.54%, 0.17% and 0.68% respectively.

Full-scale feature fusion and CBAM: Due to the rich color and wide coverage area of remote sensing images, in addition to the features of clouds and snow, a lot of feature information of other ground objects can be extracted. In the process of cloud and snow detection, too much characteristic information will lead to the dilution of cloud and snow features and affect the accuracy of cloud and snow detection. Full-scale feature fusion and CBAM can effectively reduce the dilution of cloud and snow features and strengthen the network's attention to cloud and snow. As can be seen from Table 2, mPA value, mIoU value and mPrecision value increased by 1.38%, 0.84% and 0.41% on the basis of multidimensional image input.

Weighted cross entropy loss: There is a lot of background information in the cloud and snow detection data set of remote sensing image, and the proportion of cloud and snow is relatively small. In the process of network model training, it is inevitable that the model will be biased to background information. The weighted cross entropy loss designed in this paper can effectively avoid this problem. When the number of pixels of a certain class is smaller, the weight coefficient will be larger, and the network will be better able to learn this class, in addition, it can also solve the problem of sample imbalance. As can be seen from Table 2, mPA value, mIoU value and mPrecision value are further improved. Finally, mPA value, mIoU value and mPrecision value reach 92.76%, 81.74% and 86.49% respectively.

### Comparison and analysis of algorithms

**Figure 10:**
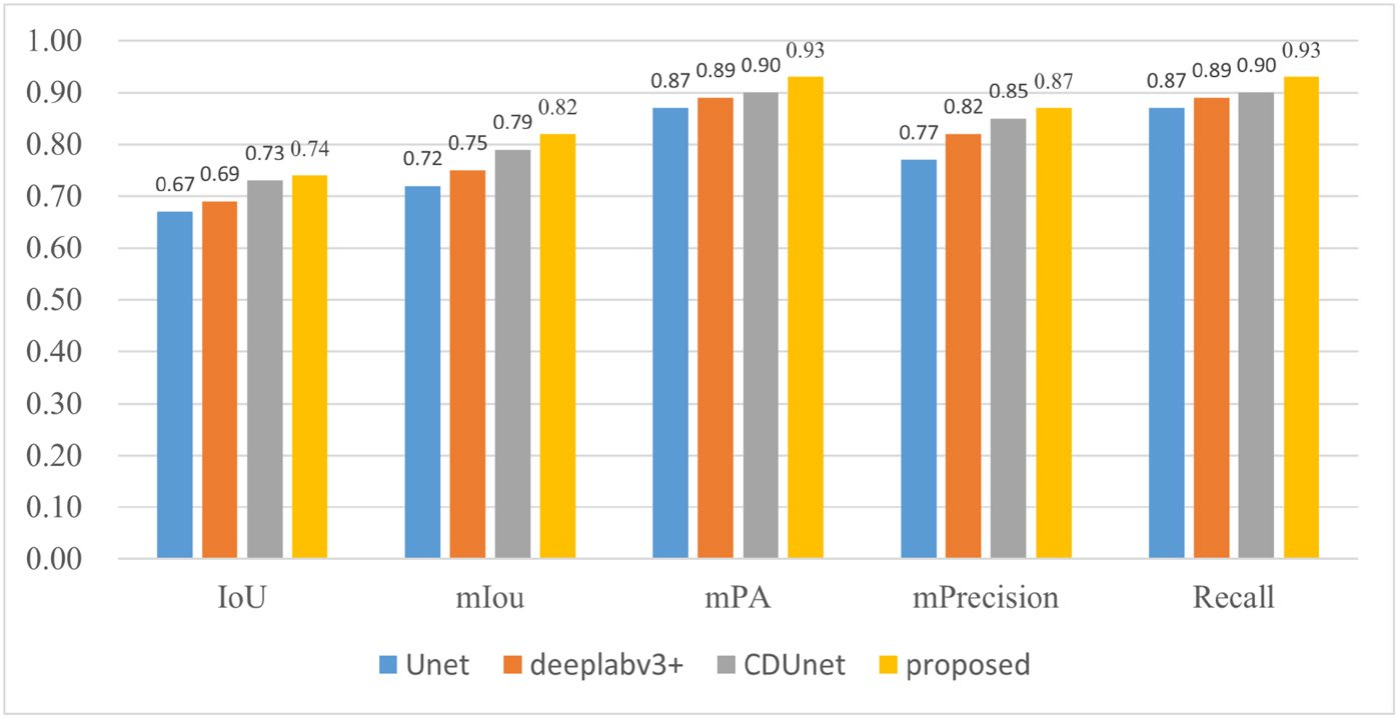
Comparison of evaluation results of IoU and other 5 quantitative indicators.

Under the constructed cloud and snow detection data set, the proposed method is compared with other cloud detection methods based on Unet^35^, Deeplabv3+^24^ and CDUnet^23^. In this paper, intersection over union (IoU), mean intersection over union (mIoU), mean pixel accuracy (mPA), mean precision (mPrecision) and recall are selected as the measurement indexes of the experimental results, and the results are shown in Fig. 10. As can be seen from the figure, the IoU value, mIoU value, mPA value, mPrecision value and recall value of this method are 0.74, 0.82, 0.93, 0.87 and 0.93 respectively, compared with other cloud detection methods, it has been improved. This is because other methods only focus on clouds in remote sensing images. For snow with very similar features to clouds, it is easy to misjudge cloud and snow due to insufficient feature information extracted.

**Figure 11:**
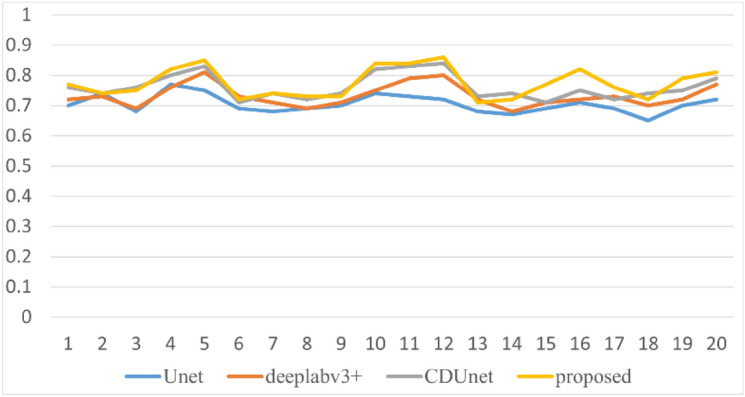
Comparison of mIoU values of different methods.

**Figure 12:**
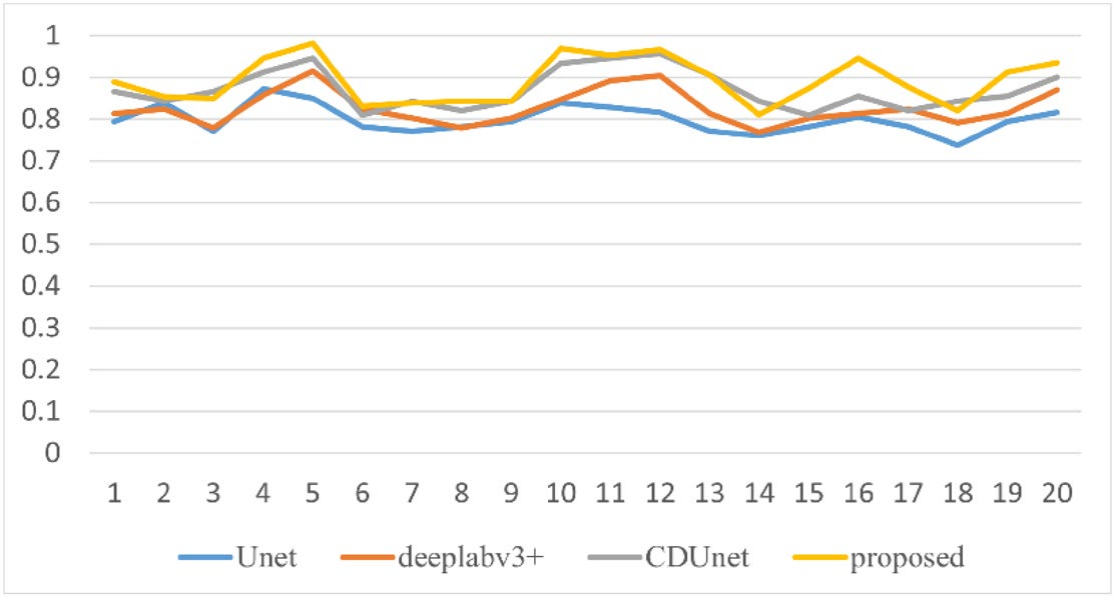
Comparison of mPA values of different methods.

In this paper, 20 remote sensing images of different landforms including plain, snow field, ocean and desert are selected from the validation set, and mIoU values and mPA values of different methods are output respectively. The results are shown in Figs. 11 and 12. As can be seen from the figure, the mIoU value and mPA value of the proposed method are generally higher than those of other methods when tested under remote sensing images of various landforms, with the mIoU value generally reaching above 0.75 and mPA value generally reaching above 0.85. There are a lot of broken clouds in 14 and 18 remote sensing images, and the cloud boundary information is complex, CDUnet network adds high-frequency feature extractor and multi-scale convolution to refine the cloud boundary, and the detection result of broken clouds is slightly better than the method proposed in this paper.

**Figure 13:**
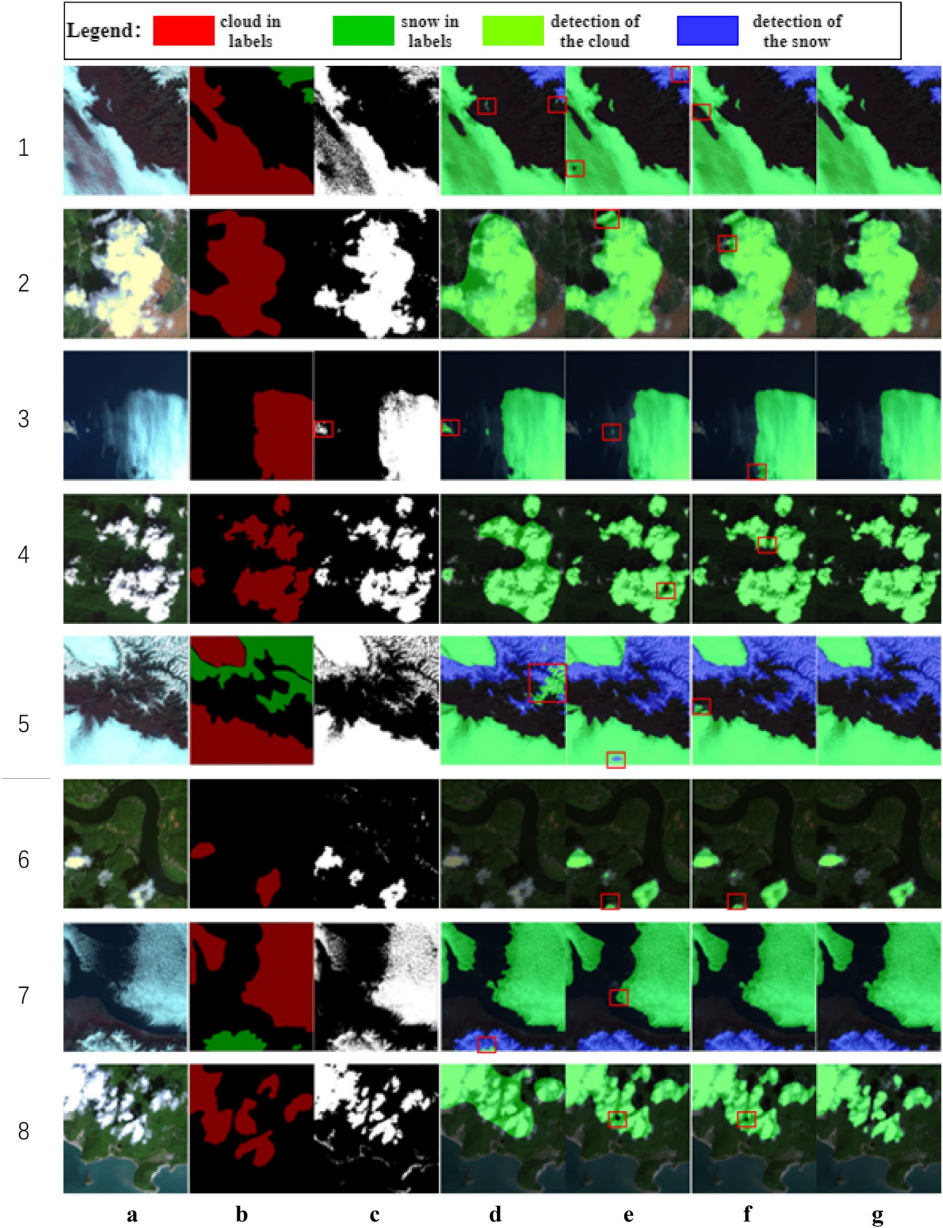
Prediction results (**a**) Original images; (**b**) Label images; (**c**) OTSU; (**d**) Unet; (**e**) Deeplabv3+; (**f**) CDUnet; (**g**) proposed.

As shown in Fig. 13, the proposed method is compared with the threshold segmentation method (OTSU) and the above neural network-based method, and the detection results are respectively output in eight landforms, including desert, town, ocean, plain, snow field and river. Figure 13c shows the detection results of OTSU. As only a single threshold value can be set, this method can detect clouds more accurately for a single remote sensing image with only clouds. However, for remote sensing images with snow and white ground objects, misdetection is serious. As shown in c3, c5 and c7, this method also misdetects white islands and snow as clouds. Figure 13d is the output result of Unet-based cloud detection method. Due to simple feature extraction, features of cloud and snow cannot be accurately distinguished, resulting in low cloud detection accuracy of this method and cloud boundary cannot be accurately located, as shown by c2. When there is interference from snow and white ground objects, it is also prone to false detection. As shown in d3, white islands are mistakenly detected as clouds, and snow in d5 is also mistakenly detected as clouds. Figure 13e shows the output of the cloud detection method based on Deeplabv3+. Due to the addition of a simple and effective decoder module, this method has a finer division of object boundary, which is greatly improved compared with Unet method. However, there will be false detection inside the cloud, and the distinction between cloud and snow is not high, as shown in e5 and e8. Figure 13f is the output of cloud detection method based on CDUnet. This method introduces high-frequency feature extractor and multi-scale convolution, which can well detect cloud and cloud shadow in the image, and has high accuracy in the distinction between cloud and snow. However, misdetection may occur at cloud junction, as shown in f4 and f5, false detection occurs at the cloud-snow junction and multi-cloud junction. Figure 13g is the output of the method in this paper, which can not only accurately detect the clouds in remote sensing images, but also distinguish the clouds and snow in images well, with few cases of missed detection and false detection.

## Conclusion

In this paper, based on Unet3+ network structure, multi-dimensional remote sensing image is used as network input through color space conversion, Resnet50 is used as feature extraction network, and CBAM is added to improve the network's attention to cloud and snow in the image, and deeply extract cloud and snow features. At the same time, the weighted cross entropy loss is designed to solve the sample imbalance problem. Experimental results show that the proposed method can effectively eliminate interference information and accurately extract cloud and snow from remote sensing images of various landforms. In the next step, we will continue to enrich the cloud detection data set, further improve the anti-interference ability against snow and other white ground objects in the cloud detection process, and explore a new lightweight deep network model to reduce computing overhead and improve the speed of cloud detection.

## Data Availability

The datasets used or analyzed during the current study are available from the corresponding author on reasonable request.

## Code availability

The codes used during the current study are available from the corresponding author on reasonable request.
